# Chemoradiotherapy in pancreatic carcinoma

**DOI:** 10.4103/0971-5851.60049

**Published:** 2009

**Authors:** Sushmita Pathy, Subhash Chander

**Affiliations:** *Department of Radiation Oncology, All India Institute Of Medical Sciences, Ansari Nagar, New Delhi – 110 029, India*

**Keywords:** *Carcinoma*, *chemoradiation*, *pancreas*

## Abstract

Pancreatic cancer patients present late in their course and surgical resection as a modality of treatment is of limited value. Majority develop loco-regional failure and distant metastasis, therefore, adjuvant therapy comprising of radiotherapy and chemotherapy are useful treatment options to achieve higher loco-regional control. Specialized irradiation techniques like intra-operative radiotherapy that help to increase the total tumor dose have been used, however, controvertible survival benefit was observed. Various studies have shown improved median and overall survival with chemoradiotherapy for advanced unresectable pancreatic carcinoma. The role of new agents such as topoisomerase I inhibitors also needs further clinical investigations.

## INTRODUCTION

Pancreatic carcinoma incidence and mortality rates have steadily increased over the last decade. It is the fourth leading cause of mortality from malignant diseases in United States, and presents particularly difficult management problems. Estimated number of new cases of pancreatic cancer and the deaths resulting from it are 33730 and 32300 respectively in the United States in 2006[1]In India the incidence varies from 0.-3.5/100,000 population, according to NCRP Biennial Report.[2]

The pancreatic carcinoma is difficult to diagnose. At presentation only 20% of the patients with adenocarcinoma of the pancreas have resectable tumors. Forty per cent of the patients have locally advanced disease while remaining 40% have metastatic disease.[3–5] The survival rates in pancreatic adenocarcinoma patients are stuck at abysmally low levels. In patients with resectable disease but positive surgical margins or with locally advanced disease, the median survival time is less than a year following chemoradiation. Two year survival rates range from 20-40% with surgery alone. The overall five-year survival rate is less than four per cent.[6]Despite resection, local recurrence and distant metastases occur in up to 50% of patients mostly within liver and peritoneum.[7,8]

On the basis of treatment failure patterns, adjuvant irradiation in both preoperative and postoperative setting along with chemotherapy has been used to reduce local recurrence and distant metastases.

The present review focuses on the various adjuvant therapy schedules for pancreatic carcinoma, current treatment principles and future prospects.

## RESECTABLE PANCREATIC CANCER

Recognition of the high rates of local, regional, and metastatic tumor recurrence following a surgical resection has prompted widespread efforts to develop effective adjuvant treatment for pancreatic cancer. Adjuvant chemoradiation in resectable pancreatic cancer has been tried in the past to improve local control and survival. In a prospective randomized trial by Gastrointestinal Tumor Study Group (GITSG)[9] the patients, after curative resection, were randomized to adjuvant radiation and chemotherapy versus observation. Twenty two patients were randomized to no adjuvant treatment while 21 to combined therapy consisting of External Beam Radiotherapy (EBRT) and 5 FU. Combined therapy consisted of 40 Gy EBRT given in two courses of 20 Gy/10 fractions each separated by an interval of two weeks. Concomitant 5 FU was administered for three consecutive days at a daily dose of 500mg/m^2^ at the beginning of each 20 Gy course. Results indicated a doubling of median and long-term survival (median 20 months vs. 11 months; two years survival 43% vs. 15%). Despite this improvement 71% of patients manifested recidivation, with 50% relapsing in the liver.

Several retrospective studies including those from Mayo Clinic,[10] John Hopkins University,[11]University of Pennsylvania[12]and France[13] have confirmed results of GITSG [Table 1].

**Table 1:** Adjuvant studies in pancreatic cancer

Adjuvant study	Study design	EBRT dose (Gy)	Chemotherapy	MS, Months	Comments
Kalser *et al*.	22 pts surgery alone	None	None	11	
GITSG (1985)	21 pts to CTRT	40	5 FU Bolus	20	
				(*P* = 0.01)
Foo *et al*.	29	45-54	5 FU Bolus	22.8	Improved survival with adjuvant therapy
Mayo Clinic (1993)					
Yeo *et al*.	53 pts surgery alone	None 40-45 split course	None 5 FU Bolus	13.5	
John Hopkins (1997)	99 pts standard	50.4-57.6 + liver 23-27	5-FU CI	21 (*P* = 0.002)	
	21 intensive			17.5	
Whittington (1991)	33 pts surgery alone	None	None	15	
	10 pts RT alone	45-54	None	15	
	28 pts CTRT	45-54	5 FU infusion	15

EBRT: External beam radiotherapy, MS: Median survival

The Mayo Clinic experience was reviewed for survival and pattern of failure after resection alone and after adjuvant treatment with chemoradiation.[9,10]Twenty nine patients underwent curative resection followed by adjuvant radiation and 5 FU chemotherapy. The median RT dose was 54 Gy; the radiation portals encompassed the tumor bed and regional node. Seventeen out of 29 patients received continuous course treatment, 27 of 29 patients received concomitant bolus 5 FU chemotherapy. Median survival was 22.8 months, with a two-year survival of 48%, similar to results achieved with adjuvant therapy in GITSG study, however, high incidence of hepatic failureand peritoneal seeding was also observed.[10,11]

Yeo *et al*. in a prospective study evaluated two difference postoperative adjuvant chemoradiaiton to no adjuvant therapy. 174 patients underwent pancreatico duodenectomy and were offered standard therapy with external beam radiotherapy (EBRT) and 5 FU Bolus, intensive therapy EBRT and 5-FU infusion with prophylactic hepatic irradiation or no ajdjuvan therapy. The authors concluded adjuvant chemoradiation significantly improves survival intensive therapy offered nosurvival advantage.^11^Yeo and coworkers performed analysis of patients who underwent pancreatic duodenectomy with or without adjuvant chemoradiation for cancer of head of pancreas at Johns Hopkins University Hospital.[14]In the initial study, statistically significant difference in survival was observed among those patients with positive margins compared with negative margins. Postoperative adjuvant chemotherapy and radiation therapy together with negative nodal status and tumor diameter smaller than 3cm significantly favored long term survival. The EORTC and GITSG[15]evaluated patients with early pancreatic carcinoma. Patients were randomized to EBRT plus concurrent 5 FU or observation alone after surgical resection. However, 20% of the patients did not receive any treatment due to postoperative complications. The two-year overall survival rates were 23% and 27% for those randomized to observation and those to adjuvant therapy. The trial had many flaws as it had a long accrual period (8 years) and very early stage cases were primarily enrolled in the trials.

Preliminary analysis of the European study group for pancreatic cancer trials -1 (ESPAC-1) comparing adjuvant chemotherapy (weekly 5 FU and Folinic acid) and RT-40 Gy with chemotherapy alone, showed no benefit for chemoradiation.[16]

A further publication has provided upgraded information of this trial. For the patients who received chemoradiotherapy the five-year survival rate was estimated to be 10% whereas among patients who were not given chemoradiotherapy it was 20% (*P* = 0.05). The five-year survival rate was 21% among patients who received chemotherapy and eight per cent among patients who did not receive chemotherapy (*P* = 0.009). Thus adjuvant chemotherapy was associated with a significant survival benefit in patients with resected pancreatic cancer, yet adjuvant chemoradiotherapy produced a deleterious effect on survival.[17]

It is difficult to draw conclusions about the role of RT in the treatment of pancreatic carcinoma from current randomized trials due to use of split course radiotherapy, low RT dose and incomplete therapy. Thus the traditional therapy for resectable tumor continues to be pancreaticoduodenectomy followed by adjuvant radiation and concomitant 5 FU based chemotherapy.

## RADIATION THERAPY

The GITSG trial discussed above, did not demonstrate a significant difference in causes of death or sites of treatment failure between the various patient groups randomized to observation or adjuvant chemoradiotherapy and radiotherapy. High local failures were observed in view of low radiation dose.

Specialized irradiation techniques that increase the dose to the tumor volume have been used to improve local tumor control without increasing normal tissue morbidity. IORT allows direct visualization and delivery of high doses of radiation to the tumor bed during the operation, a facileness not available with conventional radiation. Sensitive normal structures may be moved away from the radiation field. Intraoperative radiotherapy does not necessarily improve the survival even though local recurrences may be observed less often.

NCI conducted a trial in which 11 patients with resectable disease were randomized to IORT (20 Gy) or EBRT (45-55 Gy) and additional four patients of stage I received no further treatment.[18]Median survival was 18 months. Local recurrence was observed in 47% and regional recurrence in 47% instances. Intrabdominal metastases consisting of peritoneal seeding occurred in 45%, liver metastasis in 47% and distant metastases in 62%.

Similar results were reported by Fossati *et al*.[19] in their review of 33 patients who underwent resection for pancreatic carcinoma plus IORT with or without EBRT with or without chemotherapy. The local recurrence rate was significantly lower with IORT (25% vs. 55.8%); overall survival however was not significantly different in the IORT group compared with control group.

Zerbi and colleagues[20]also reported on use of IORT in patients with operable pancreas cancer. IORT (12.5 to 20 Gy) was delivered after surgery in 43 patients. Forty seven additional patients underwent resection alone. Local recurrence was significantly decreased in IORT group (27% vs. 56%, *p*< 0.01). Thus IORT may benefit patients with resectable disease, but has little impact on survival[21,22] However with the advances of systemic therapy, this may confer a significant benefit in long-term outcome. Yamaguchi *et al*. clued that only the combination of IORT with external radiotherapy led to some betterment in the short-term results of patients with resectable pancreatic carcinoma. These authors felt it was advisable to give external radiotherapy after pancreatectomy and IORT.[23]

More recently, Japanese workers have reported a study evaluating hyperfractionated accelerated radiotherapy (HART) and 5-FU and cisplatin in patients with unresectable pancreatic carcinoma. HART was given as 1.5 Gy twice daily doses, separated by six hours for five days a week (total dose of 45 Gy). They observed that 35% patients showed partial response, 50% remained stable while another 15% had local progressive disease. The authors found the toxicity to be well tolerated and local efficacy of the treatment was as per their expectations.[24]

## CHEMOTHERAPY

Whittington *et al*[12]in a retrospective analysis of 70 patients with pancreatic cancer divided therapy to no adjuvant therapy (33 patients), postoperative Rtalone or plus bolus5 FU chemotherapy (19 patients) 8/19 patients received bolus 5 FU and 96 hour infusion5 FU chemotherapy with RT (20 patients). Median survival was similar; however, three-year survival was significantly improved with chemo-sensitized irradiation.

Gemcitabine is also a potent radiation sensitizer of human pancreatic cancer cells and was found to be superior to bolus 5 FU in a prospective randomized trial.[25] Trials are underway to evaluate its role in the preoperative setting. A large phase III trial with 5 FU was performed by Eastern cooperative Oncology Group (ECOG)[26] coparing single agent gemcitabine (162 pts) with gemcetabine plus 5 FU (160 pts) median survival 6.7 months combination arm and 5.4 months with single agent.

In a multicenter study, Rocha Lime *et al*.[27] compared the overall survfival associated with Irinotecan and gemcitabine (IRINOGEM) versus Gemcitabine (GEM) a single agent concluded IRIMNOGEM safely improved tumour response rate compared with GEM, but did not alter overall survival.

Heinemann *et al*. compared Gemcitabine and cisplatin combination with Gemcitabine alone. Combination therapy arm was accompanied by a prolonged median progression-free survival (5.3 months vs. 3.1 months). The median overall survival was also prolonged in the former group. Albeit tumor response rates were comparable in the two treatment arms (10.2% vs. 8.2%).[28] These studies are summarized in Table 2.

**Table 2:** Chemotherapy in pancreatic carcinoma

Authors	No. of patients	Study design	Median survival	Comments
Heinemann *et al*. (2006)	195	Gemcitabine	6	Combination are /	
		Gemcitabine + cisplatin	7.5	was more effective no statistical significance
Berlin *et al*.	160	Gemcite + 5FU	6.7	
ECOG (2002)	162	Gemcitabine	5.4	
Rocha Lima	360	Gem	6.6	Irinotican safety
(2004)		Gem + Irinotecan	6.3	improved tumor response but did not alter overall survival

## PREOPERATIVE VS. POSTOPERATIVE THERAPY

The timing of adjuvant therapy has been a subject of debate. Preoperative radiation therapy offers the advantage of tumor down staging, decrease in the risk of tumor seeding during surgery and avoiding treatment delays in postoperative setting due to postoperative complications.

In a trial conducted by ECOG, preoperative 5 FU, Mitomycin C and EBRT were administered in patients with resectable tumors. Two years survival was 27%.[29,30]

Pendurthi *et al*.[31] while comparing preoperative and postoperative chemoradiation, observed no significant difference in survival or local control; however, treatment delay of more than 60 days was observed in postoperative group in 22% patients. Quite recently, Massucco *et al*. admitted that the indications of pancreatic resection subsequent to chemo-radiation are not described clearly. These workers subjected 28 patients with locally advanced pancreatic carcinoma to Gemcitabine-based chemoradiotherapy. Patients showing partial response or a stable disease were operated upon. The results thus obtained were compared to another group of 44 cases, resected for localized cancer (irrespective of adjuvant treatment status). Median survival was 15.4 months and 14 months respectively. It was concluded that transition of an unresectable lesion to a resectable one is a rarity in patients having pancreatic cancer.[32]

## PROPHYLACTIC LIVER IRRADIATION

More than half of the patients treated with surgery and adjuvant therapy develop hepatic metastases. Phase I-II trials evaluating prophylactic hepatic irradiation was conducted. Komaki *et al*.[33] from Medical College of Wisconsin conducted a pilot study in which 15 patients were treated with EBRT consisting of 61.2 Gy to pancreas and 23.4 Gy to liver,5 FUwas given with EBRT and for 12 months after RT. Two years disease free survival was 46.7%. Two patients had hepatic metastasis as first site of failure.

Evans *et al*.[34]in an analysis of 11 patients treated with 50.4 Gy external beam RT to the pancreas with concurrent continuous infusion of5 FU and hepatic irradiation up to 23.4 Gy on day 8 to 21, resectable patients were taken up for pancreato-duodenectomy and additional 10 Gy was delivered as IORT to the tumor bed. The study suggested the lack of benefit to prophylactic hepatic irradiation and was terminated in view of two treatment related deaths.

## UNRESECTABLE PANCREATIC CANCER

Approximately 40-50% of the tumors are classified as locally advanced disease and their median survival is 3-5 months.[35]As primary surgical approach is not possible in these cases, systemic treatment alone or in combination with radiotherapy is necessary, recent research has been focused on intensification of RT and chemotherapy.

O'Conner *et al*. reported 77 patients subjected to attempted surgical resection and IORT for pancreatic or periampullary adenocarcinoma. Twenty-four patients had unresectable tumors and went through surgical bypass and IORT. Patients with unresectable disease treated with bypass and IORT had a median survival of 11 months only. The authors concluded that IORT is well tolerated. They observed that patients with periampullary adenocarcinoma had a better prognosis than those with pancreatic adenocarcinoma. Obviously the patients with unresectable pancreatic disease fared worse.[36]

## CHEMORADIATION

Combined chemoradiation therapy for unresectable carcinoma of the pancreas was first studied at Mayo clinic.[37] Thirty two patients with unresectable adenocarcinoma of pancreas were randomized to receive EBRT (35 to 40 Gy with four weeks) with or without 5 FU based chemotherapy. Results showed a statistically significant improvement in median survival with addition of 5 FU, 10.4 months vs 6.3 months. GITSG[38,39] published results of three arm trial in which patients were randomized to receive external beam RT to 60 Gy alone, external beam RT to 40 Gy plus bolus 5 FU or external beam RT 60 Gy plus bolus 5 FU. Patients treated with combined modality therapy had an improvement in median survival (42 vs. 22 weeks *P*< 0.01) compared with those treated with RT alone. No significant difference was noted in outcome with treatment by 40 Gy or 60 Gy.

On the basis of the results of this study, combined chemoradiotherapy became the standard treatment in locally unresectable pancreatic cancer. A randomized prospective trial evaluating 5 FU alone versus 5 FU plus EBRT (40 Gy) was performed by ECOG.[40] Ninety one patients were entered. Median survival was 8.2 months for patients receiving chemotherapy alone and 8.3 months for combined modality therapy.

The benefit of chemo-irradiation was studied by GITSG[41]and it was observed that both median and overall survival rates were improved with addition of radiotherapy to chemotherapy. The treatment schedules were Streptozocin, Mitomycin and 5-fluorouracil (SMF) versus radiation and 5-fluorouracil followed by SMF combination chemotherapy. Median survival for the combined-modality treatment was 42 weeks. This is better than the 32 weeks median survival with chemotherapy alone.

Hyperfractionated radiotherapy was evaluated in a pilot study by Gastrointestinal Tumor Study Group. EBRT consisting of 50.4 Gy (1.2 Gy twice daily, four to six hours apart) was given along with 5 FU on the first three and last three days of radiation therapy. Further chemotherapy was given using Stretozotocin and Mitomycin and 5 FU. One-year survival rate was 39% and median survival was 35 weeks.[42]

Abe and colleagues[43]published the largest experience with Intraoperative Radiotherapy (IORT) for unresectable pancreatic carcinoma. One hundred and three patients were treated with surgery plus EBRT, intraoperative radiotherapy or combination of both EBRT consisting of 55-60 Gy and IORT ranging from 25-40 Gy depending on the tumor site. Median survival was nine months vs. 12 months vs. 5.5 months to surgery plus EBRT, IORT plus EBRT and IORT or surgery alone respectively. Ma et studied the effect of Interferon-α for its synergistic effects with chemoradiation in pancreatic adenocarcinoma and concluded that IFN-α has direct cytotoxic effects acting as radiosensitizer and controls tumor re-growth after cisplatin therapy.[44]

Ghassan *et al*. treated 175 patients having locally advanced or metastatic pancreatic adenocarcinoma with Exatecan plus Gemcitabine. Exatecan Mesylate is a hexacyclic, water-soluble, topoisomerase-1 inhibitor. Another 174 patients were given Gemcitabine alone. The median survival time was 6.7 and 6.2 months respectively (*P* = 0.52). They concluded that Exatecan along with Gemcitabine was not found superior to Gemcitabine alone.[45]

## QUALITY OF LIFE CONSIDERATIONS

Neoptolemos *et al*.[17]in the European Study Group for Pancreatic Cancer Trials1 evaluated quality of life in these patients. Questionnaires regarding the quality of life were filled by 152 of the 289 patients. There were no significant differences in the post- resection quality of life within 12 months between patients who received chemotherapy and those not receiving chemotherapy (*P* = 0.75) and also between the patients who received chemoradiotherapy and those who did not receive chemoradiotherapy (*P*=0.17).

## CONCLUSION

Pancreatic carcinoma is a leading cause of death. These tumors present late in their course and less than 20% are amenable to surgical resection. The treatment of pancreatic cancer continues to be a challenge to the oncologists.

Emerging techniques in the fields of surgery, radiotherapy, chemotherapy and immunotherapy offer hope for greater locoregional control, survival and quality of life for this highly fatal disease. There is a need for improvement in local control for both resectable and unresectable disease, and the evolution of three-dimensional and intensity modulated techniques offers the potential for more precise targeting of the primary lesion and thus may allow safe escalation of tumor dose.

Adjuvant chemotherapy with Gemcitabine has recently demonstrated better survival outcomes

Newer chemotherapeutic drugs including topoisomerase I inhibitors, thymidylate synthatase inhibitors and angiogenesis inhibitors, matrix metalloproteinase inhibitors, interleukin 2 may have the potential to counter the high regional and distant relapse.[46]

Further understanding of pancreatic tumor biology may lead to optimized treatment selection. Advances in imaging and molecular genetics will help in early diagnosis and more effective novel treatment.
